# Ovarian superstimulation and in vivo embryo production using a short or long-term progesterone treatment and different porcine FSH presentations in acyclic Toggenburg goats

**DOI:** 10.1007/s11250-026-05016-7

**Published:** 2026-04-11

**Authors:** Ana Paula S. Cupello, Priscila Del’Aguila-Silva, Paulo Sergio C. Rangel, Luciana V. Esteves, Felipe Z. Brandão, Joanna M. G. Souza-Fabjan, Jeferson F. Fonseca

**Affiliations:** 1 Faculdade de Veterinária, Universidade Federal Fluminense, Niterói, RJ Brazil; 2 Embrapa Caprinos e Ovinos, Coronel Pacheco, MG Brazil

**Keywords:** MOET, Porcine follicle-stimulating hormone (pFSH), Superovulation, Non-surgical embryo recovery (NSER), Goats

## Abstract

This 2 × 2 factorial study evaluated different progesterone treatment durations and porcine follicle-stimulating hormone (pFSH) presentations for superovulation in acyclic Toggenburg goats. Goats (*n* = 56) received short (six days, G6) and long-term (17 days, G17) progesterone intravaginal devices combined with 133 mg (G6^133mg^ and G17^133mg^) or 250 IU of pFSH (G6^250IU^ and G17^250IU^) administered in six decreasing doses from 48 h before to 12 h after device removal. Does were naturally mated while in estrus. To monitor ovulation and the number of corpora lutea, ovarian transrectal ultrasound evaluations were performed on the days of the first pFSH administration, device removal, and six days later. Non-surgical embryo recovery (NSER) was performed 6–7 days after the first breeding. The G6^250IU^ group had a shorter estrus duration and required fewer matings than the other groups (*P* < 0.05). Overall, the interval between the last dose of pFSH and the first sign of estrus detected (46.2 ± 2.3 vs. 35.3 ± 2.5 h) and the duration of estrus (38.2 ± 1.9 vs. 31.3 ± 3 h) differed (*P* < 0.05) between FSH^133mg^ and FSH^250IU^, respectively, regardless of progesterone treatment duration. Shorter duration of NSER proceeding and higher embryo viability rate (*P* < 0.05) were observed in G17 groups. However, the percentage of viable structures was higher (*P* < 0.05) in G6^133mg^ (52.5%; 21/40) and G17^250IU^ (54.5%; 30/55) groups than in the G6^250IU^ (10.6%; 5/47) and G17^133mg^ (23.3%; 7/30) groups. In conclusion, short-term treatment with 133 mg of pFSH is effective in achieving satisfactory embryo viability rate while minimizing the animal’s exposure to progesterone.

## Introduction

Goat production is an important component of global livestock systems, supplying meat, milk, fiber, and leather (Nakafeero et al. 2024). The global goat population has grown by about 17% over the last decade (FAOSTAT 2023), largely driven by the growing economic profitability of goat farming in developing countries (Luo et al. 2019) and by advances in genetic improvement and reproductive biotechnologies (Simões et al. 2021). Among these technologies, multiple ovulation and embryo transfer (MOET) remains the most widely used tool to accelerate genetic gain in small ruminants (Souza-Fabjan et al. 2021, 2023), although its efficiency is still limited by variability in superovulatory responses and embryo yield, highlighting the need for protocol optimization (Bartlewski 2019).

To ensure the success of MOET, synchronization of the follicular wave and ovulation is crucial and can be achieved through ovulation induction protocols (Khan et al. 2023). Progestogen-based protocols are well-established for this purpose and are capable of inducing estrus/ovulation during seasonal anestrus when combined with gonadotropins (Fonseca et al. 2017). These protocols typically use intravaginal devices for short-, medium-, or long-term periods of time (Pietroski et al. 2013; Gore et al. 2020). Additionally, an adequate progesterone profile prior to embryo recovery may determine the non-surgical embryo recovery (NSER) success (Figueira et al. 2020). Nevertheless, large individual variation persists in ovarian response to superovulation (Fonseca et al. 2022), which is influenced by the type, origin, purity, LH: FSH (luteinizing hormone: follicle-stimulating hormone) ratio, dose, and administration schedule of FSH (Mikkola and Taponen 2017; Gutiérrez-Reinoso et al. 2022; Khan et al. 2023).

In cattle, high LH concentrations during early follicular wave growth can impair follicular maturation, ovulation, and superovulatory response (Mikkola and Taponen 2017; Redhead et al. 2018), yet no studies have compared different FSH presentations in goats. Another challenge is reducing hormonal exposure while maintaining, or improving, embryo yield. Shorter progesterone protocols may improve welfare, lower costs, and reduce progesterone-associated vaginitis (Simões et al. 2024), but their efficacy in acyclic goats remains unclear. Moreover, the combined effects of progesterone-exposure length and FSH formulation on superovulation and in vivo embryo production have not been simultaneously assessed in this species.

Considering the aforementioned gaps, we hypothesized that short-term progesterone protocols could match or even surpass long-term protocols in superovulatory response and embryo yield while minimizing hormone exposure. Thus, the objective of this study was to evaluate two durations of intravaginal progesterone device use (6 vs. 17 days) combined with two FSH presentations (133 mg vs. 250 IU) on estrus expression, ovarian response, embryo collection procedure, and in vivo embryo yield in acyclic Toggenburg goats.

## Materials and methods

### Ethics approval, location, and general conditions

This research was approved by the local Animal Care Committee (UFF / 0116–2011) and was conducted in full compliance with the guidelines of the Brazilian Society of Laboratory Animal Science, ensuring the well-being of the animals involved. This is a 2 × 2 factorial study carried out during the non-breeding season (July-August) at the Embrapa Dairy Cattle experimental field station, in Coronel Pacheco, Minas Gerais State, Brazil (latitude 21º35’S and longitude 43º15’W).

Before synchronization, the absence of corpora lutea (CL) was confirmed for two consecutive estrous cycles. Fifty-six clinically healthy, multiparous, non-pregnant, non-lactating, acyclic Toggenburg goats were enrolled, with a mean body weight of 61.4 ± 7.6 kg, body condition score of 3.1 ± 0.3 (scale 1 to 5, 1 = emaciated and 5 = obese), and mean age of 5.7 ± 1.3 years. Animals were housed in group pens and fed corn silage plus balanced concentrate, with free access to mineralized salt (Caprinofós^®^ Tortuga, São Paulo, Brazil) and water.

### Ovulation induction and superovulation protocols

**Fig. 1:**
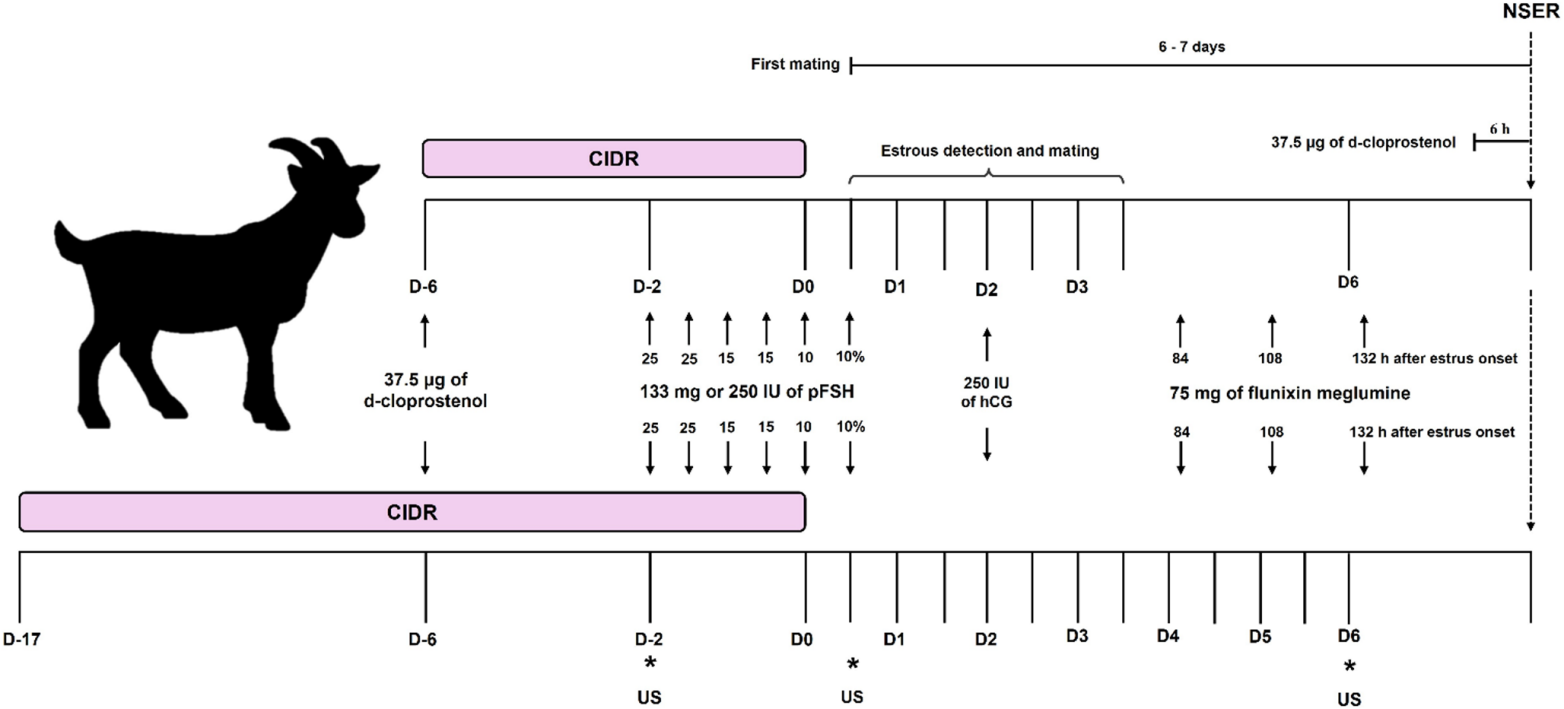
Schematic presentation of study design. Short- and long-term superovulation protocols tested in Toggenburg goats. CIDR^Ⓡ^: controlled internal drug release; pFSH: porcine follicle-stimulating hormone; hCG: human chorionic gonadotropin; NSER: non-surgical embryo recovery; US: transrectal ultrasound evaluations

The goats were randomly assigned to short-term (6 days; G6, *n* = 28) or long-term (17 days; G17, *n* = 28) progesterone-based ovulation induction protocols (Fig. 1). Protocols began during the anovulatory period with insertion of an intravaginal progesterone-releasing device (0.33 g P_4_; CIDR^®^, Zoetis) on D-6 (G6) or D-17 (G17), for synchronization of the follicular growth wave, with device removal defined as Day 0 (D0). All animals received 37.5 µg d-cloprostenol i.m. (Prolise^®^, Agener União, São Paulo, Brazil) on D-6. Superovulation started 48 h before device removal using either a total dose of 133 mg (Folltropin-V^®^, Vetoquinol, São Paulo, Brazil; G6^133mg^ and G17^133mg^; *n* = 14 animals per group) or 250 IU (Pluset^®^, Hertape Calier, Barcelona, Spain; G6^250IU^ and G17^250IU^; *n* = 14 animals per group) of porcine FSH (pFSH), administered intramuscularly in six decreasing doses at 12 h intervals (25-25-15-15-10 and 10% of the total dose).

At 36 h after device removal, all goats received 250 IU human chorionic gonadotropin (hCG; Vetecor 5000, Ceva, Juatuba, Brazil) i.m. Estrus detection began 12 h after device removal and continued at 12 h intervals for 60 h. Six fertile adult bucks (female: male ratio 4:1) were used for natural mating; each receptive doe was mated at least twice at 12 h intervals. To standardize anti-inflammatory support during early luteal development, all goats received three doses of 75 mg flunixin meglumine (Flumax, JA Saúde Animal, Patrocínio Paulista, Brazil) i.m. at 84, 108, and 132 h after estrus onset.

### Ovarian ultrasonography

Transrectal B-mode ultrasound evaluations were performed by the same experienced operator at three time points: first pFSH administration, 12 h after device removal, and six days post-device removal to verify ovulation and quantify the CLs. Ovulations were defined as the presence of a large follicle(s) recorded at the time of the previous ultrasonographic examination and confirmed by the detection of the CLs on the day of embryo collection.

The Mindray Vet (Mindray DP 3300 Vet, Shenzhen, China) ultrasound equipment was used with a multifrequential linear transrectal transducer (7.5 MHz), coupled to a plastic extender to allow its manipulation and equipment configurations were standardized to all examinations: the settings transducer frequency of 7.5 MHz, overall gain set to 80%, a depth of 6.2 cm, and a dynamic range of 70 dB. Animals were restrained in a quadrupedal position, and acoustic gel was used. After transrectal introduction, the uterus and ovaries were located, and detected CLs were quantified (Rodriguez et al. 2019).

### Cervical relaxation and NSER procedure

Embryo recovery was performed 6–7 days after estrous onset. Cervical dilatation was induced with 37.5 µg of d-cloprostenol i.m. given 6 h before embryo flushing. The NSER was performed according to Fonseca et al. (2018): through a prior cervical immobilization and traction, followed by transposition of the cervix with a catheter and uterine flushing. All animals were submitted to Embrapa’s protocol of analgesia and anesthesia receiving 0.1 mg/kg of 1% acepromazine (Acepran 1%^®^, Vetinil/Univet, São Paulo, Brazil) i.m. and dipyrone and hyoscine solution (Buscofin Plus^®^; Agener União-Saúde Animal, São Paulo, Brazil) given at doses up to 5 mL i.m. and 5 mL i.v. 20 min before the procedure, along with an epidural block, using 2 mL of 2% lidocaine hydrochloride into the sacrococcygeal space immediately before introducing the vaginal speculum, and local cervical anesthesia, by pushing a sterile gauze soaked with 5 mL of 2% lidocaine into the cervical opening (Fonseca et al. 2022). The recovered structures were morphologically evaluated using a stereomicroscope (SMZ 445, Nikon, Tokyo, Japan) at 20 to 50X magnification. The embryos were classified according to the International Embryo Transfer Society criteria for embryo viability as grades I to III for transferable-quality embryos, or grade IV for embryos with late development and/or morphologically degenerated.

### Data analysis

The following variables were recorded (mean ± SEM or %): Estrous response (%); Interval to estrus (time elapsed between the administration of the last dose of pFSH and the detection of the first sign of estrus; h); Duration of estrus (h); Matings per female; Animals ovulating (%); Number of corpora lutea per female; Responsive donors (≥ 3 CL) (%); Duration of collection procedure (min); Recovery rate (%; number of recovered structures / number of CL); Fluid recovery efficiency; Total structures recovered per female; Females with embryos recovered (%); Viable embryos per female [number of structures - (number of degenerated) - (number of unfertilized)]; Unfertilized oocytes per female; Degenerate structures per female; Embryo viability rate (%; number of viable embryos recovered / number of structure recovered); Freezable embryos per female (embryos grade I and II / females collected); Morulae (%, number of morulae / number of females collected); Blastocyst (%, number of blastocyst/number of females collected); Females with more embryos than the average (%).

Statistical analysis was performed using the software BioEstat 5.3 (Belém, Brazil) and IBM SPSS Statistics software, version 19. The Shapiro-Wilk and Levene tests were used to verify normality and homogeneity of variances, respectively. Non-parametric data were analyzed by the Mann-Whitney test and the Kruskall-Wallis followed by Dunn’s post hoc; for parametric data, t-test and ANOVA followed by Tukey’s post hoc was applied. Frequencies were assessed by Chi-square or Fisher’s exact test. Interval and estrus duration parameters were also analyzed by Pearson’s and Spearman’s correlation tests, applying the former to variables within normal range and the latter to those that did not follow normal distribution. Interaction effects between treatments were not investigated due to limitations in the dataset, particularly the reduced number of animals per treatment group, which would result in insufficient statistical power to reliably estimate interaction terms. Differences were considered significant when *P* < 0.05, while 0.1 > *P* > 0.05 was considered a tendency. Results were presented as mean ± standard error of the mean (SEM).

## Results

Regardless of the experimental group, 78.6% (44/56) of the animals responded to hormonal protocol and presented estrus, while 75% (42/56) of animals had ovulation detected during ovarian ultrasonography, with an average number of CLs per ovulating female of 4.9 ± 0.5. One female was removed from the study due to health problems unrelated to the present study, and therefore it was only possible to collect its data up to the ovulation detection time. Among ovulating goats, 75.6% (31/41) presented three or more CLs, being considered as responsive donors.

The NSER procedure was successfully accomplished in 100% (41/41) of the goats. Across treatments, the overall recovery rate was 46.4% (172/371) among all animals, with a fluid recovery efficiency of 97.4 ± 0.5%. Embryos were recovered from 43.9% (18/41) of flushed females, yielding on average 4.1 ± 0.7 structures recovered, 1.8 ± 0.5 viable embryos, 2.3 ± 0.5 unfertilized oocytes, 0.3 ± 0.1 degenerated structures, 1.2 ± 0.4 freezable embryos, 1.0 ± 0.3 morulae, and 0.5 ± 0.2 blastocyst per female. The data for the main treatments groups and the individual categories are presented in Tables 1 and 2, respectively.

**Table 1:** Variables (mean ± SEM or %) for estrus behavior expression, matings, superovulatory response and embryo recovery of grouped totals by term and dosage in acyclic Toggenburg goats undergone to short (six days, Total G6) or long-term (17 days, Total G17) treatment with progesterone releasing device, followed by 133 mg (Total^133mg^) or 250 IU (Total^250IU^) of porcine FSH

Variables	Grouped totals by term			Grouped totals by dosage		
	Total G6	Total G17	*P*-value	Total^133mg^	Total^250IU^	*P*-value
Estrus response (%)	82.1 (23/28)	75 (21/28)	0.74	78.6 (22/28)	78.6 (22/28)	1.00
Interval to estrus (h)	44.2 ± 2.3	37 ± 2.9	0.12	46.2 ± 2.3 ^A^	35.3 ± 2.5 ^B^	0.004
Duration of estrus (h)	32.3 ± 2.8	37.3 ± 2.2	0.31	38.2 ± 1.9 ^A^	31.3 ± 3 ^B^	0.03
Matings per female	2.6 ± 0.2	3.0 ± 0.2	0.15	3.0 ± 0.2	2.5 ± 0.2	0.13
Animals ovulating (%)	75 (21/28)	75 (21/28)	1.00	75 (21/28)	75 (21/28)	1.00
Number of corpora lutea per female	5.6 ± 0.7	4.1 ± 0.6	0.12	4.7 ± 0.7	5 ± 0.6	0.44
Responsive donors (≥ 3 CLs) (%)	80 (16/20)	71.4 (15/21)	1.00	70 (14/20)	81 (17/21)	0.59
Duration of collection procedure (min)	34.3 ± 2.1 ^A^	25.6 ± 2.4 ^B^	0.003	31.8 ± 2.5	28 ± 2.2	0.50
Recovery rate (%)	43.7 (87/199)	49.4 (85/172)	0.32	42.7 (70/164)	49.2 (102/207)	0.25
Fluid recovery efficiency (%)	97.2 ± 0.8	97.6 ± 0.8	0.58	97.1 ± 0.9	97.7 ± 0.6	0.78
Total structures recovered per female	4.4 ± 1.1	4 ± 0.9	0.77	3.5 ± 0.9	4.9 ± 1.1	0.56
Females with embryos recovered (%)	30 (6/20)	57.1 (12/21)	0.15	50 (10/20)	38.1 (8/21)	0.65
Viable embryos per female	1.3 ± 0.6	1.8 ± 0.6	0.40	1.4 ± 0.3	1.6 ± 0.4	0.92
Unfertilized oocytes per female	2.7 ± 0.7	1.8 ± 0.7	0.19	1.7 ± 0.6	2.7 ± 0.8	0.30
Degenerated structures per female	0.4 ± 0.2	0.5 ± 0.2	0.68	0.4 ± 0.2	0.5 ± 0.2	0.68
Embryo viability rate (%)	29.9 (26/87)	43.5 (37/85)	0.08	40 (28/70)	34.3 (35/102)	0.55
Freezable embryos per female	1 ± 0.5	1.3 ± 0.5	0.44	1.1 ± 0.5	1.2 ± 0.5	0.77
Morulae	0.7 ± 0.3	1.3 ± 0.6	0.42	0.8 ± 0.3	1.2 ± 0.6	0.98
Blastocyst	0.6 ± 0.4	0.5 ± 0.2	0.53	0.6 ± 0.4	0.5 ± 0.2	0.53

_Note: A,B Different letters within a row indicate significant differences (*P* < 0.05)_

**Table 2:** Variables (mean ± SEM or %) for estrus behavior expression, matings, superovulatory response and embryo recovery in acyclic Toggenburg goats treated with intravaginal progesterone releasing device for six (G6) or 17 (G17) days and submitted to superovulatory treatment with different presentations of porcine FSH, such as 133 mg (G6^133mg^ or G17^133mg^) or 250 IU (G6^250IU^ or G17^250IU^) of pFSH

Variables	G6^133mg^	G6^250IU^	G17^133mg^	G17^250IU^	*P*-value
Estrus response (%)*	85.7 (12/14)	78.6 (11/14)	71.4 (10/14)	78.6 (11/14)	-
Interval to estrus (h)	48 ± 3 ^A^	40 ± 3.2 ^AB^	44 ± 3.8 ^A^	30.5 ± 3.4 ^B^	0.004
Duration of estrus (h)	38.7 ± 2.6 ^A^	25.5 ± 4.3 ^B^	37.6 ± 2.9 ^A^	37.1 ± 3.5 ^A^	0.03
Matings per female	3 ± 0.2 ^B^	2.1 ± 0.3 ^A^	3.0 ± 0.2 ^B^	3.0 ± 0.3 ^B^	0.04
Animals ovulating (%)*	78.6 (11/14)	71.4 (10/14)	71.4 (10/14)	78.6 (11/14)	-
Number of corpora lutea per female	5.3 ± 1.1	5.9 ± 0.9	4.1 ± 1	4.2 ± 0.7	0.53
Responsive donors (≥ 3 CLs) (%)*	70 (7/10)	90 (9/10)	70 (7/10)	72.7 (8/11)	-
Duration of collection procedure (min)	34.6 ± 3.4 ^A^	34 ± 2.5 ^B^	28.9 ± 3.7 ^AB^	22.5 ± 2.8 ^B^	0.03
Recovery rate (%)	43 (40/93)	44.3 (47/106)	42.3 (30/71)	54.5 (55/101)	0.29
Fluid recovery efficiency (%)	96.6 ± 1.2	97.9 ± 0.9	97.7 ± 1.3	97.5 ± 1	0.81
Total structures recovered per female	4 ± 1.6	4.7 ± 1.4	3 ± 0.8	5 ± 1.6	0.93
Females with embryos recovered (%)*	40 (4/10)	20 (2/10)	60 (6/10)	54.5 (6/11)	-
Viable embryos per female	2.1 ± 1.1	0.5 ± 0.3	0.7 ± 0.3	2.7 ± 1.1	0.43
Unfertilized oocytes per female	1.7 ± 0.9	3.6 ± 1.2	1.7 ± 0.9	1.9 ± 1.2	0.27
Degenerated structures per female	0.2 ± 0.1	0.6 ± 0.4	0.6 ± 0.3	0.4 ± 0.3	0.84
Embryo viability rate (%)	52.5 (21/40) ^A^	10.6 (5/47) ^B^	23.3 (7/30) ^B^	54.5 (30/55) ^A^	< 0.0001
Freezable embryos per female	1.8 ± 1	0.2 ± 0.2	0.4 ± 0.3	2.2 ± 0.8	0.23
Morulae	0.9 ± 0.5	0.5 ± 0.3	0.7 ± 0.3	1.8 ± 1.1	0.79
Blastocyst	1.2 ± 0.9	0 ± 0	0 ± 0	0.9 ± 0.4	0.26

_Note: * Descriptive data between treatments_

_A, B Different letters within a row indicate significant differences (*P* < 0.05)_

When treatments were analyzed individually (Table 1), differences (*P* < 0.05) were observed for: interval and duration of estrus, with an average of 40.7 ± 1.9 h and 34.7 ± 1.8 h, respectively, among all animals; number of matings per female (2.8 ± 0.1); duration of collection procedure (29.8 ± 1.7 min); and embryo viability rate (36.6%; 63/172).

There was no correlation between the interval and duration of estrus across the treatments used throughout the study *(P* > 0.05). However, in relation to the total grouped by dosage in females submitted to superovulation protocol with 250 IU, it was possible to observe a weak correlation between these two variables, in which the longer the estrus interval, the shorter the duration of estrus presented by does (*p* = 0.0798; Spearman’s coefficient = -0.3814).

## Discussion

To the best of our knowledge, this study is the first to simultaneously evaluate varying progesterone exposure durations and two pFSH presentations in acyclic goats. Although the estrus induction rate (78.6%) was consistent across treatments, it fell below the 85–100% typically reported for small ruminants using intravaginal devices (Fonseca et al. 2022; Dias et al. 2023), as detailed in Table 3. While the absence of functional corpora lutea at d-cloprostenol administration minimizes luteolytic variability (Martinez-Ros et al. 2018), seasonal anestrus likely hindered the response. During the non-breeding season, suppressed LH pulsatility and heightened estradiol negative feedback limit behavioral expression (Fatet et al. 2011). In acyclic females, estrus depends strictly on exogenous progesterone withdrawal and efficient follicular steroidogenesis; consequently, minor variations in wave emergence or LH activity can impair outcomes (Menchaca and Rubianes 2004). Additionally, the lack of body-weight-adjusted FSH dosing may have led to inconsistent follicular stimulation, which collectively explains the lower rates observed in our study.

**Table 3:** Comparison of results from estrus synchronization protocols, superovulation, and in vivo embryo production in goats submitted to non-surgical embryo recovery

Breed	Estrus synchronization protocol	Superovulation protocol	Estrus response (%)	Number of corpora lutea per female	Recovery rate (%)	Number of viable embryos per female		Embryo viability rate (%)	Reference	
Toggenburg	6 days of 0.33 g CIDR + 37.5 µg d-cloprostenol	133 mg pFSH (six doses) + 250 IU hCG	85.7	5.3 ± 1.1	43.0		2.1 ± 1.1	52.5	Present study	
		250 IU pFSH. (six doses) + 250 IU hCG	78.6	5.9 ± 0.9	44.3		0.5 ± 0.3	10.6		
	17 days of 0.33 g CIDR + 37.5 µg d-cloprostenol	133 mg pFSH (six doses) + 250 IU hCG	71.4	4.1 ± 1	42.3		0.7 ± 0.3	23.3		
		250 IU pFSH. (six doses) + 250 IU hCG	78.6	4.2 ± 0.7	54.5		2.7 ± 1.1	54.5		
Saanen	11 days of 60 mg medroxyprogesterone acetate + 50 µg cloprostenol	200 mg pFSH (six doses)	85.0	11.5 ± 6	53.2		ND	88.0	Lima-Verde et al. (2003)	
Toggenburg	11 days of 60 mg medroxyprogesterone acetate + 37.5 µg d-cloprostenol	200 mg pFSH (six doses)	100 (0% urea diet)	ND	ND		7.0 ± 2.12	100.0	Amorim et al. (2011)	
			80 (2.4% urea diet)	ND	ND		5.4 ± 3.3	84.4		
Alpine	11 days of 0.3 g CIDR + 75 µg of d-cloprostenol	133 mg pFSH (six doses)	100.0	12.8 ± 3.2	65.1		5.2 ± 0.4	ND	Batista et al. (2014)	
		133 mg pFSH (six doses) + 4.8 µg/kg BW of human recombinant leptin	100.0	12.2 ± 3.9	65.7		6.8 ± 0.4	ND		
Moxotó and Canindé	6 days of 60 mg medroxyprogesterone acetate + 100 µg sodium cloprostenol	133 mg pFSH (six doses)	90.0	11.9 ± 2.1	45.8		4.9 ± 1.7	89.6	Fonseca et al. (2022)	
Canindé	10 days of 60 mg of medroxyprogesterone acetate + 50 µg d-cloprostenol	120 mg pFSH (five doses)	100.0	13.7 ± 1.6	86.8 ± 5.6		11.2 ± 1.5	ND	Souza-Fabjan et al. (2022)	

BW: body weight; CIDR: controlled internal drug release; i.m.: intramuscularly; pFSH: porcine follicle-stimulating hormone; hCG: human chorionic gonadotropin; eCG: equine chorionic gonadotropin; GnRH: gonadotropin-releasing hormone; ND: not-determined

The biological impact of FSH presentation depends on the FSH: LH ratio and extraction origin (Rodriguez et al. 2019). Although 133 mg of Folltropin-V^®^ and 250 IU of Pluset^®^ are often considered equivalent in FSH activity, Pluset contains significantly higher LH concentrations. High LH levels during superstimulation can trigger premature follicular luteinization, accelerated follicular turnover, and compromised oocyte competence (Mikkola and Taponen 2017). This mechanism likely explains the shorter estrus duration and reduced mating opportunities observed in the 250 IU (Pluset) groups in the present study. While excessive LH can increase ovulation rates, it often impairs embryo quality due to asynchronous oocyte maturation (D’Alessandro and Martemucci 2016). According to Rodriguez et al. (2019), the effect of altering the FSH/LH ratio in superovulatory protocols on ovulatory rate is controversial and appears to vary across breeds. They observed that ewes superovulated with 100 mg of pFSH showed more adequate development of luteal structures than those treated with 200 mg. Notably, in our study, these LH-driven behavioral changes did not alter CL numbers or the total number of recovered structures, suggesting that the endocrine imbalance at these doses was sufficient to affect behavior but not ovulatory output.

The overall number of CL and embryo yield in the present study was lower than that reported in some previous goat superstimulation studies. Fonseca et al. (2022) reported an average of 11.9 ± 2.1 CL/female, associated with 4.9 ± 1.7 viable embryos per female of Moxotó and Canindé breeds. Meanwhile, during the breeding season, Amorim et al. (2011) obtained 7.0 ± 2.12 viable embryos per flushed Toggenburg goats. It suggests a suboptimal superovulatory response in our study, linked to the donors’ metabolic and endocrine status. Nonetheless, this study was conducted during the goats non-breeding season, which may be a factor that undermines the results obtained. Another critical factor is the significantly higher body weight of the Toggenburg does used in this experiment. Since pFSH was administered in fixed doses, heavier females likely experienced a lower effective concentration of circulating gonadotropins due to a higher volume of distribution. This diluted hormonal stimulus may have failed to adequately support recruitment of the antral follicle pool, leading to fewer preovulatory follicles and, consequently, lower ovulatory rates (Rodriguez et al. 2019).

Despite 100% NSER success in the present study, the duration of the embryo collection procedure was shorter in G17 group than in G6 group (26 vs. 34 min), which differs from Oliveira et al. (2022), whose group with a long and short-term protocol had similar collection durations (31 vs. 28 h, respectively). More prolonged exposure to progesterone, as observed in the present study in G17, might lead to physiological changes in females that contribute to cervical dilation, facilitating the NSER procedure. These changes occur due to the accumulation of enzymes and molecules, such as arachidonic acid, used in prostaglandin F2α (PGF2α) synthesis, as well as estrogen-stimulated expression of oxytocin receptors, as postulated earlier (Figueira et al. 2020). The application of d-cloprostenol in cervical dilation protocols for NSER results in the release of oxytocin, which, in conjunction with PGF2α, relaxes the cervix, facilitating the passage of the catheter through the cervical rings and reducing the time required to perform the NSER procedure, as previously reported by Figueira et al. (2020).

The duration of P4 exposure and the FSH presentation did not influence the proportion of females with recovered embryos, yet they significantly affected the embryo viability rate in our study. A central and novel finding of the present study is the superior performance of the groups G6^133mg^ and G17^250IU^, which showed the highest embryo viability. In the G6^133mg^ group, the combination likely promoted a more synchronized cohort of recruitable follicles, limiting premature LH exposure during the critical window of final follicular growth (Maciel et al. 2019; Fonseca et al. 2022). By minimizing LH bioactivity, this endocrine setting may have favored a gradual acquisition of oocyte competence, resulting in embryos with higher developmental potential. Conversely, in the G17^250IU^ group, sustained P4 concentrations are known to suppress premature LH pulsatility and stabilize the intrafollicular environment during the follicular wave, thereby preventing early luteinization and supporting coordinated follicular growth before deviation (Bartlewski et al. 2017). Oliveira et al. (2014) and Oliveira et al. (2022) also reported no difference in recovery rate between short (6 days) and long-term (12 days) protocols. However, unlike our study, the authors also found no difference in embryo viability between the groups.

Additionally, since the use of intravaginal progesterone devices is associated with vaginitis and high costs (Simões et al. 2024), opting for hormonal treatments with short progesterone exposure is more advisable for health and reproductive management efficiency (Khan et al. 2023). By minimizing the duration of exogenous progesterone influence, these protocols not only mitigate inflammatory risks but also simplify animal handling without compromising the efficiency of estrus induction or superovulatory outcomes.

All these findings suggest a similarity between long and short exposure regarding the evaluated parameters, indicating a preference for short protocols due to previously demonstrated factors. However, the interaction observed here emphasizes that FSH presentation cannot be interpreted independently from P4 exposure, and that optimizing superstimulation protocols in goats may require tailoring FSH dose (and LH content) to the endocrine profile created by different synchronization strategies, and also highlights the need for further studies comparing different superovulation protocols, especially the ones exploring a body weight-dependent pFSH dose.

In conclusion, the duration of exposure to progesterone and the type of gonadotropin determined effects on embryo production, whereby both short-term exposure to progesterone combined with 133 mg of pFSH (G6^133mg^) and long-term exposure combined with 250 IU of pFSH (G17^250IU^) produced the highest embryo viability rates among the tested protocols in acyclic Toggenburg goats. Although overall superovulatory response and embryo yield did not differ markedly among treatments, the short-term protocol associated with 133 mg pFSH stands out as a practical and efficient alternative. This approach minimizes progesterone exposure, reduces handling and management time, and may lower costs without compromising embryo viability. Taken together, the results indicate that refining superovulation protocols by adjusting progesterone duration and the presentation of pFSH can contribute to more efficient and animal-friendly MOET programs in dairy goats.

## Data Availability

The data that support the findings of this study are available from the corresponding author upon reasonable request.

## References

[CR1] Amorim LS, Torres CAA, Siqueira LGB, Fonseca JF, Guimarães JD, Carvalho GR, Alves NG, Oliveira MMNF (2011) Embryo development and follicular status of Toggenburg does fed urea diet. Rev Bras Zootec 40:277–285. 10.1590/S1516-35982011000200007

[CR3] Bartlewski PM (2019) Recent advances in superovulation in sheep. Rev Bras Reprod Anim 43:126–128

[CR2] Bartlewski PM, Sohal J, Paravinja V, Baby T, Oliveira MEF, Murawski M, Schwarz T, Zieba DA, Keisler DH (2017) Is progesterone the key regulatory factor behind ovulation rate in sheep? Domest Anim Endocrinol 58:30–38. 10.1016/j.domaniend.2016.06.006 27639459 10.1016/j.domaniend.2016.06.006

[CR4] Batista AM, Gomes WA, Carvalho CC, Monteiro PL Jr, Silva FL, Almeida FC, Soares PC, Carneiro GF, Guerra MM (2014) Effect of leptin on in vivo goat embryo production. Reprod Domest Anim 49(3):476–480. 10.1111/rda.12314 24731188 10.1111/rda.12314

[CR5] D’Alessandro AG, Martemucci G (2016) Superovulatory response to gonadotrophin FSH/LH treatment and effect of progestin supplement to recipients on survival of transferred vitrified embryos in goats. Theriogenology 85(2):296–301. 10.1016/j.theriogenology.2015.09.038 26483311 10.1016/j.theriogenology.2015.09.038

[CR6] Dias JH, Vergani GB, Gonçalves JD, Oliveira TA, Penitente-Filho JM, Pereira VSA, Esteves SN, Garcia AR, Batista RITP, Oliveira MEF, Souza-Fabjan JMG, Fonseca JF (2023) Different doses of pFSH are effective to promote follicular growth, superovulatory response, and embryo yield in White Dorper ewes. Small Rumin Res 220:06914. 10.1016/j.smallrumres.2023.106914

[CR7] FAO – Food and Agriculture Organizations of the United Nations (2023) Database. Crops and livestock products. FAOSTAT. http://fao.org/faostat/en/#data. Accessed 07 de December 2025

[CR8] Fatet A, Pellicer-Rubio MT, Leboeuf B (2011) Reproductive cycle of goats. Anim Reprod Sci 124(3–4):211–219. 10.1016/j.anireprosci.2010.08.029 20888155 10.1016/j.anireprosci.2010.08.029

[CR9] Figueira LM, Alves NG, Souza-Fabjan JMG, Oliveira MEF, Lima RR, Souza GN, Fonseca JF (2020) Preovulatory follicular dynamics, ovulatory response and embryo yield in Lacaune ewes subjected to synchronous estrus induction protocols and non-surgical embryo recovery. Theriogenology 145:238–246. 10.1016/j.theriogenology.2019.11.004 31753477 10.1016/j.theriogenology.2019.11.004

[CR10] Fonseca JF, Souza-Fabjan JMG, Oliveira MEF, Cruz RC, Esteves LV, Matos de Paiva MPSL, Brandão FZ, Mancio AB (2017) Evaluation of cervical mucus and reproductive efficiency of seasonally anovular dairy goats after short-term progestagen-based estrous induction protocols with different gonadotropins. Reprod Biol 17:363–369. 10.1016/j.repbio.2017.10.002 29031924 10.1016/j.repbio.2017.10.002

[CR11] Fonseca JF, Oliveira MEF, Brandão FZ, Batista RITP, Garcia AR, Bartlewski PM, Souza-Fabjan JMG (2018) Non-surgical embryo transfer in goats and sheep: the Brazilian experience. Reprod Fertil Dev 31:17–26. 10.1071/RD18324 32188539 10.1071/RD18324

[CR12] Fonseca JF, Vergani GB, Lima MSD, Silva KM, Monteiro AWU, Ramos AF, Alves BRC, Souza-Fabjan JMG, Oliveira MEF, Batista RITP (2022) Nonsurgical embryo recovery as a feasible tool for supporting embryo biobanks of locally adapted Brazilian sheep and goats. Biopreserv Biobank 20:493–501. 10.1089/bio.2021.0066 34747654 10.1089/bio.2021.0066

[CR13] Gore DLM, Mburu JN, Okeno TO, Muasya TK (2020) Short-term oestrous synchronisation protocol following single fixed-time artificial insemination and natural mating as alternative to long-term protocol in dairy goats. Small Rumin Res 192:106207. 10.1016/j.smallrumres.2020.106207

[CR14] Gutiérrez-Reinoso MA, Aguilera CJ, Navarrete F, Cabezas J, Castro FO, Cabezas I, Sanchez O, Garcia-Herreros M, Rodriguez-Alvarez L (2022) Effects of extra-long-acting recombinant bovine FSH (bscrFSH) on cattle superovulation. Animals 12:153. 10.3390/ani12020153 35049777 10.3390/ani12020153 PMC8772581

[CR16] Khan S, Jamal MA, Khan IM, Ullah I, Jabbar A, Khan NM, Liu Y (2023) Factors affecting superovulation induction in goats (*Capra hericus*): An analysis of various approaches. Front Vet Sci 10:1152103. 10.3389/fvets.2023.1152103 37035816 10.3389/fvets.2023.1152103 PMC10079885

[CR17] Lima-Verde J, Lopes Junior E, Teixeira D, Paula N, Medeiros A, Rondina D, Freitas V (2003) Transcervical embryo recovery in Saanen goats. S Afr J Anim Sci 33(2):127–131. 10.4314/sajas.v33i2.3766

[CR18] Luo J, Wang W, Sun S (2019) Research advances in reproduction for dairy goats. Asian-Australas J Anim Sci 32:1284–1295. 10.5713/ajas.19.0486 31357269 10.5713/ajas.19.0486 PMC6668861

[CR19] Maciel GS, Rodriguez MGK, Santos VJC, Uscategui RAR, Nociti RP, Maronezi MC, Oliveira CS, Feliciano MAR, Vicente WRR, da Fonseca JF, Oliveira MEF (2019) Follicular dynamics and in vivo embryo production in Santa Inês ewes treated with smaller doses of pFSH. Anim Reprod Sci 209:106137. 10.1016/j.anireprosci.2019.106137 31514927 10.1016/j.anireprosci.2019.106137

[CR20] Martinez-Ros P, Lozano M, Hernandez F, Tirado A, Rios-Abellan A, López-Mendoza MC, Gonzalez-Bulnes A (2018) Intravaginal device-type and treatment-length for ovine estrus synchronization modify vaginal mucus and microbiota and affect fertility. Anim (Basel) 8226. 10.3390/ani8120226 10.3390/ani8120226 PMC6316288 30501021

[CR21] Menchaca A, Rubianes E (2004) New treatments associated with timed artificial insemination in small ruminants. Reprod Fertil Dev 16(4):403 – 13. 10.10371/RD04037. PMID: 15315739 10.10371/RD04037 15315739

[CR22] Mikkola M, Taponen J (2017) Embryo yield in dairy cattle after superovulation with Folltropin or Pluset. Theriogenology 88:84–88. 10.1016/j.theriogenology.2016.09.052 27865416 10.1016/j.theriogenology.2016.09.052

[CR23] Nakafeero A, Gonzalez-Bulnes A, Martinez-Ros P (2024) Use of short-term CIDR-based protocols for oestrus synchronisation in goats at tropical and subtropical latitudes. Animals 14:1560. 10.3390/ani14111560 38891607 10.3390/ani14111560 PMC11171354

[CR24] Oliveira ME, Feliciano MA, D’Amato CC, Oliveira LG, Bicudo SD, Fonseca JF, Vicente WR, Visco E, Bartlewski PM (2014) Correlations between ovarian follicular blood flow and superovulatory responses in ewes. Anim Reprod Sci 144:30–37. 10.1016/j.anireprosci.2013.10.012 24290565 10.1016/j.anireprosci.2013.10.012

[CR26] Oliveira MEF, Arrais AM, de Mello MRB, Vergani GB, Figueira LM, Esteves SN, Pereira VSDA, Garcia AR, Bartlewski PM, da Fonseca JF (2022) Study of the factors affecting embryo yields and quality in superovulated Morada Nova ewes that underwent non-surgical uterine flushing. Reprod Domest Anim 57:393–401. 10.1111/rda.14077 34967972 10.1111/rda.14077

[CR27] Pietroski ACCA, Brandão FZ, Souza JMG, Fonseca JF (2013) Short, medium or long-term hormonal treatments for induction of synchronized estrus and ovulation in saanen goats during the nonbreeding season. Rev Bras Zootec 42:168–173. 10.1590/S1516-35982013000300004

[CR28] Redhead AK, Siew N, Lambie N, Carnarvon D, Ramgattie R, Knights M (2018) The relationship between circulating concentration of AMH and LH content in the follicle stimulating hormone (FSH) preparations on follicular growth and ovulatory response to superovulation in water buffaloes. Anim Reprod Sci 188:66–73. 10.1016/j.anireprosci.2017.11.010 29175175 10.1016/j.anireprosci.2017.11.010

[CR29] Rodriguez MGK, Maciel GS, Uscategui RAR, Santos VJC, Nociti RP, Silva PDA, Feliciano MAR, Brandão FZ, Fonseca JF, Oliveira MEF (2019) Early luteal development in Santa Inês ewes superovulated with reduced doses of pFSH. Reprod Domest Anim 54:456–463. 10.1111/rda.13374 30421465 10.1111/rda.13374

[CR31] Simões J, Abecia JA, Cannas A, Delgadillo JA, Lacasta D, Voigt K, Chemineau P (2021) Review: Managing sheep and goats for sustainable high yield production. Animal 15:100293. 10.1016/j.animal.2021.100293 34294548 10.1016/j.animal.2021.100293

[CR32] Simões ML, Brandão FZ, Santos JDR, Pinto PHN, Schmidt APP, Laeber CCR, Knust ND, Rodriguez MGK, Fila D, Vázquez MI, Ungerfeld R (2024) Combining two injectable progesterone formulas for estrous synchronization in ewes. Anim Reprod 21:e20240073. 10.1590/1984-3143-AR2024-0073 39421263 10.1590/1984-3143-AR2024-0073 PMC11486456

[CR33] Souza-Fabjan JMG, Batista RITP, Correia LFL, Paramio MT, Fonseca JF, Freitas VJF, Mermillod P (2021) In vitro production of small ruminant embryos: latest improvements and further research. Reprod Fertil Dev 33:31–54. 10.1071/RD20206 38769678 10.1071/RD20206

[CR34] Souza-Fabjan JMG, Batista RITP, Melo LM, Oliveira MAL, Chaves MS, Fonseca JF, Freitas VJF (2022) Transcervical versus laparotomy embryo recovery: What strategy is best for embryo bank formation in the Canindé goat conservation program? Biopreserv Biobank 20(2):204–207. 10.1089/bio.2021.0041 34491078 10.1089/bio.2021.0041

[CR35] Souza-Fabjan JMG, Leal GR, Monteiro CAS, Batista RITP, Barbosa NO, Freitas VJF (2023) In vitro embryo production in small ruminants: what is still missing? Anim Reprod 20:e20230055. 10.1590/1984-3143-AR2023-0055 38025995 10.1590/1984-3143-AR2023-0055 PMC10681138

